# Trends and projections of the disease burden of vitamin A deficiency in China from 1990 to 2021

**DOI:** 10.3389/fnut.2025.1633788

**Published:** 2025-08-20

**Authors:** Feng Chen, Zixue Gu, Zheng Shi, Shiyun Li, Rong Peng

**Affiliations:** ^1^School of Basic Medical Sciences and School of Nursing, Chengdu University, Chengdu, Sichuan, China; ^2^Clinical Medical College and Affiliated Hospital of Chengdu University, Chengdu, Sichuan, China

**Keywords:** vitamin A deficiency, disease burden, trend analysis, prediction, BAPC model

## Abstract

**Objective:**

The current systematic research on the disease burden of Vitamin A deficiency in China is limited. To analyze the trends in the disease burden of Vitamin A Deficiency (VAD) in China from 1990 to 2021 and predict future trends from 2022 to 2050, providing a scientific basis for the prevention of VAD in China.

**Methods:**

Based on the Global Burden of Disease (GBD) data, we extracted incidence, prevalence, and disability-adjusted life years (DALYs) for VAD in China from 1990 to 2021. The Joinpoint regression model was used to analyze temporal trends, calculating the estimated annual percentage change (EAPC) and its 95% confidence interval (CI). The Bayesian Age-Period-Cohort (BAPC) model was employed to integrate age, period, and cohort effects for predicting China’s VAD disease burden from 2022 to 2050.

**Results:**

From 1990 to 2021, the Age-Standardized Incidence Rate (ASIR), Age-Standardized Prevalence Rate (ASPR), and Age-Standardized Disability-Adjusted Life Year Rate (ASR-DALYs) of VAD in China showed a significant downward trend, with Estimated Annual Percentage Change (EAPC) values of −5.31 (95% CI: −5.65 to −4.96), −5.31 (95% CI: −5.66 to −4.96), and −3.86 (95% CI, −4.84 to −2.86), respectively. The disease burden was higher in females than in males and higher in children than in adults. According to the BAPC model, the ASR-DALYs of VAD in China are expected to stabilize from 2022 to 2050, with a gradual decline from 3. 17/100,000 in 2022 to 2.70/100,000 in 2050. The ASIR and ASPR are projected to continue declining, with ASIR decreasing from 1,838. 15/100,000 in 2022 to 269.57/100,000 in 2050 and ASPR decreasing from 1,835.08/100,000 in 2022 to 267.89/100,000 in 2050.

**Conclusion:**

While China’s VAD burden has shown sustained reduction, continued attention is needed for children and women to address the “hidden hunger” of micronutrient deficiencies. Future efforts should strengthen nutritional interventions and health education to further mitigate the disease burden.

## 1 Introduction

Vitamin A deficiency (VAD) is one of the most prominent public health issues in developing countries (1). Among various micronutrient deficiencies, VAD remains one of the major contributors to the disease burden of mortality in children under 5 years of age globally and is one of the four major nutritional deficiencies worldwide (2). Its pathophysiological mechanism involves retinol metabolism disorders, which can cause delayed growth and development, night blindness, dry cornea, and impaired immune function in children, thereby increasing the incidence and mortality of infectious diseases (3–9). In addition, recent studies have also revealed the potential association between VAD and cognitive dysfunction as well as mental illnesses (10–12), further highlighting its significance in the global disease burden. In the global burden of disease assessment, vitamin A deficiency (VAD) is identified as the second leading risk factor, with its associated disability-adjusted life years (DALYs) serving as a crucial quantitative metric for evaluating the burden of malnutrition (13). Despite a substantial decline in incidence over the past decade, vitamin A deficiency (VAD) remained the most severe subcategory of global nutritional deficiencies in 2019 (14). As the most populous country in the world, the effectiveness of VAD prevention and control in China is directly related to the achievement of the national health goals. Since the 1990s, the Chinese government has implemented nationwide nutritional improvement programs, promoted vitamin A supplements, and strengthened health education through various intervention measures (15, 16), striving to reduce the disease burden of VAD. However, despite the overall downward trend in disease burden, the epidemiological characteristics of VAD still show significant gender and age differences.

Currently, systematic research on the long-term dynamic changes and future trends of the disease burden of VAD in China is still limited. Existing literature mostly focuses on cross-sectional analysis or short-term trend assessment, lacking systematic time series studies based on national data. Therefore, based on the data released by the Global Burden of Disease Study (GBD) in 2021, this study aims to systematically analyze core indicators such as incidence, prevalence, and disability-adjusted life years (DALYs) of VAD, reveal the distribution characteristics of VAD across different genders and age groups from 1990 to 2021 and their changes over time, and use the Bayesian Age-Period-Cohort Model (BAPC) to predict the disease burden from 2022 to 2050. Identifying key intervention populations, the study hopes to provide a scientific basis for the evaluation of the prevention and control effects of VAD in China and the adjustment of strategies and measures. This will promote precise nutritional interventions and the realization of the Health China 2030 goals.

## 2 Materials and methods

### 2.1 Data sources

The data for this study were obtained from the GBD 2021 database.^1^ Based on the study objectives, the following data were extracted from the GBD database: Under “GBD Estimate,” select “Cause of death or injury”; under “Measure,” select “Incidence,” “DALYs,” and “Prevalence”; under “Metric,” select “Number,” “Percent,” and “Rate”; under “Cause,” select “Vitamin A deficiency”; under “Location,” select “China”; under “Age,” select age groups from “0–6 days” to “95 + years” in 5-year intervals; under “Sex,” select “Female,” “Male,” and “Both”; under “Year,” select all years from 1990 to 2021; and finally, click “Download” to obtain the data. The database provides epidemiological data on VAD in China from 1990 to 2021, covering core indicators such as incidence, prevalence, and DALYs to assess the trends in the disease burden of VAD. The uncertainty intervals (UI) provided by the GBD database were used to represent the confidence intervals (CI) of the data, ensuring the reliability of the study results.

### 2.2 Statistical analysis

R software version 4.1.3 was used for statistical analysis, and the ggplot2 package was used to plot trend graphs. The main analysis indicators included Age-Standardized Incidence Rate (ASIR), Age-Standardized Prevalence Rate (ASPR), Age-Standardized Disability-Adjusted Life Year Rate (ASR-DALYs), and Estimated Annual Percentage Change (EAPC). The 95% CI ofEAPC was used to determine trend changes: if the lower limit of the 95% CI of EAPC is greater than 0, the Age-Standardized Rate (ASR) shows an upward trend; if the upper limit is less than 0, the ASR shows a downward trend. The study analyzed the current status of the disease burden of VAD in China in 2021 and the trends from 1990 to 2021 across the entire population, different age groups, and genders. The BAPC model was used to predict the future trends of the disease burden of VAD in China from 2022 to 2050 based on historical data from the GBD database from 1990 to 2021. This model integrates age, period, and cohort effects, providing a scientific basis for predicting future changes in the disease burden.

## 3 Results

### 3.1 Disease burden and trends of VAD in China from 1990 to 2021

**Figure 1:**
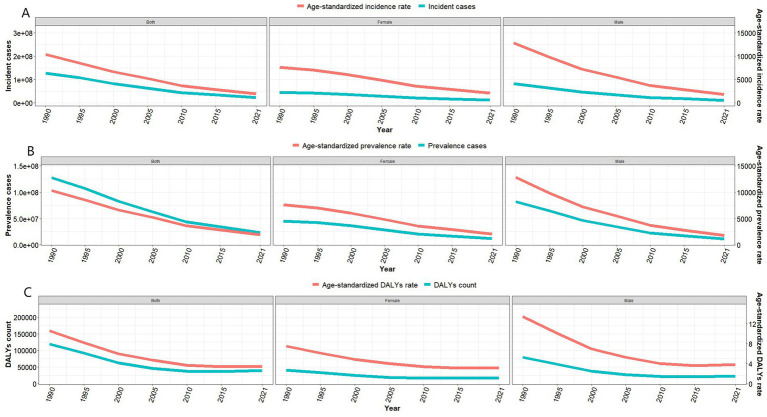
Disease burden of vitamin A deficiency in China from 1990 to 2019. **(A)** Number of VAD cases and Age-Standardized Incidence Rate; **(B)** Number of prevalent cases and Age-Standardized Prevalence Rate; **(C)** Disability-adjusted life years and Age-Standardized DALY Rate.

**Figure 2:**
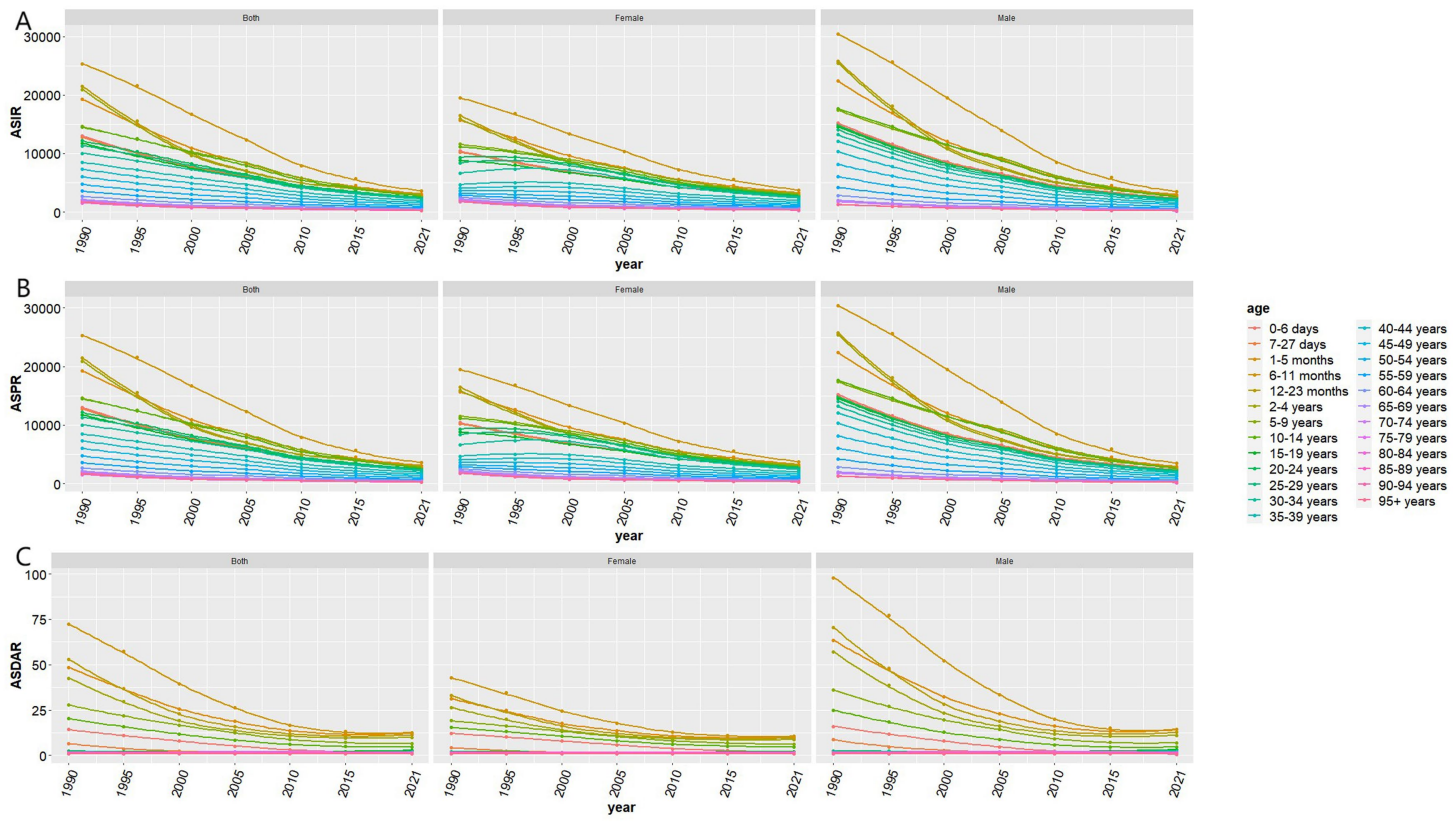
Disease burden of vitamin A deficiency across different age groups in China from 1990 to 2021. **(A)** ASIR, Age-Standardized Incidence Rate; **(B)** ASPR, Age-Standardized Prevalence Rate; **(C)** ASDAR, Age-Standardized DALY Rate.

From 1990 to 2021, the epidemiological burden of Vitamin A Deficiency (VAD) in China has significantly decreased (Figure 1, Table 1). The total number of incident cases showed a downward trend, decreasing from 127,618,000 to 23,195,000. The Age-Standardized Incidence Rate (ASIR) dropped from 10,360.50 per 100,000 in 1990 to 1951 per 100,000 in 2021, with an overall downward trend (Estimated Annual Percentage Change (EAPC) = −5.31, 95% Confidence Interval (CI) = −5.65 to −4.96) (Figure 1A). Concurrently, the total number of prevalent cases also decreased, from 127,594,000 to 23,158,000. The Age-Standardized Prevalence Rate (ASPR) decreased from 10,358.60 per 100,000 in 1990 to 1,947.50 per 100,000 in 2021, showing an overall downward trend (EAPC = −5.31, 95% CI = −5.66 to −4.96) (Figure 1B). The Disability-Adjusted Life Years (DALYs) count generally showed a downward trend, stabilizing after 2010, decreasing from 119,560.80 to 39,094.40. The Age-Standardized Disability-Adjusted Life Year Rate (ASR-DALYs) decreased from 10.60 per 100,000 in 1990 to 3.40 per 100,000 in 2021, showing a general slow downward trend, stabilizing after 2010 (EAPC = −3.86, 95% CI = −4.84 to −2.86) (Figure 1C). Stratified analysis showed that the largest decrease in ASIR was observed in the 12–23 month age group (EAPC = −6.28, 95% CI = −6.85 to −5.71) and ASPR (EAPC = −6.29, 95% CI = −6.86 to −5.72), while the most significant decrease in ASR-DALYs was in the 0–6 day age group (EAPC = −8.00, 95% CI = −8.67 to −7.33) (Figure 2).

**Table 1:** Trends in the disease burden of vitamin A deficiency in China from 1990 to 2021.

Characteristics	Incident cases No. × 10^5^(95% UI)	ASIR per 100,000 No.(95% UI)	Prevalence cases No. × 10^5^(95% UI)	ASPR per 100,000 No.(95% UI)	DALYs cases No. (95% UI)	ASR-DALYs per 100,000 No. (95% UI)
1990	1276.18 (1104.44, 1467.26)	10360.50 (9052.80, 11842.1)	1275.94 (1104.25, 1467.08)	10358.60 (9051.00, 11840.40)	119560.80 (76104.00, 176187.80)	10.60 (6.80, 15.60)
2021	231.95 (201.23, 268.81)	1951.00 (1673.00, 2272.00)	231.58 (200.89, 268.47)	1947.50(1669.70, 2268.70)	39094.40 (24872.80, 57158.00)	3.40 (2.10,5.10)
EAPC (95% CI)		−5.31 (−5.65, −4.96)		−5.31 (−5.66, −4.96)		−3.86 (−4.84, −2.86)

ASIR, Age-Standardized Incidence Rate; ASPR, Age-Standardized Prevalence Rate; ASR-DALYs, Age-Standardized DALY Rate; EAPC, Estimated Annual Percentage Change.

### 3.2 Analysis of VAD disease burden by gender and age in 2021

**Figure 3:**
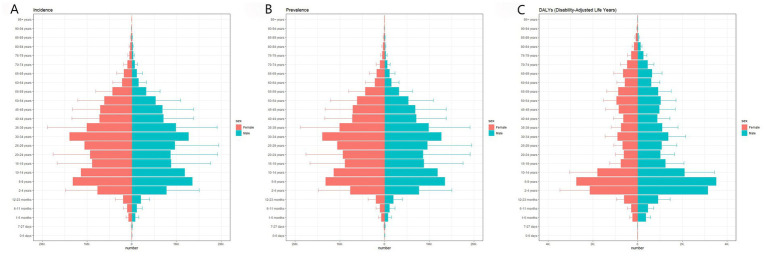
Age distribution of vitamin A deficiency disease burden in China in 2021. **(A)** Number of VAD cases by gender; **(B)** Number of prevalent cases by gender; **(C)** Disability-adjusted life years by gender.

In 2021, both the number of new cases and the number of prevalent cases of Vitamin A Deficiency (VAD) showed a fluctuating trend with age, peaking in the 5-9-year-old and 30-34-year-old groups, and then decreasing with increasing age. Disability-Adjusted Life Years (DALYs) began to rise rapidly from the 1-5-month-old age group, peaked in the 5-9-year-old group, and then gradually decreased with age (Figure 3). The Age-Standardized Incidence Rate (ASIR), Age-Standardized Prevalence Rate (ASPR), and Age-Standardized Disability-Adjusted Life Year Rate (ASR-DALYs) all generally increased and then decreased with age, reaching their peaks in the 6-11-month-old age group. The ASIR and ASPR slowly declined with age, while the ASR-DALYs rapidly decreased and stabilized after the 20-24-year-old age group (Table 2).

**Table 2:** Disease burden of vitamin A deficiency in China by gender and age in 2021.

Characteristics	Incident cases No. x10^4^ (95% UI)	ASIR per 100,000 No. (95% UI)	DALYs cases No. (95% UI)	ASR-DALYs per 100,000 No. (95% UI)	Prevalence cases No. x10^4^ (95% UI)	ASPR per 100,000 No.(95% UI)
Sex						
Both	2,319.45	1,951.00	39,094.40	3.40	2,315.85	1,947.50
	(2,012.26, 2,688.13)	(1,673.00, 2,272.00)	(24,872.8, 57,158.00)	(2.10, 5.10)	(2,008.94, 2,684.67)	(1,669.70, 2,268.70)
Female	1,192.51	2,094.30	16,871.20	3.10	1,191.34	2,091.90
	(983.44, 1,421.08)	(1,719.60, 2,542.30)	(10,725.00, 24,595.80)	(1.90, 4.60)	(982.43, 1,419.73)	(1,716.80, 2,540.20)
Male	1,126.94	1,821.10	22,223.20	3.80	1,124.50	1,816.60
	(878.17, 1,445.14)	(1,411.60, 2,309.30)	(14,005.80, 33,194.20)	(2.30, 5.60)	(875.99, 1,442.64)	(1,406.80, 2,304.50)
Age						
0–6 days	0.52	2468.00	2.40	1.20	0.51	2468.00
	(0.29, 0.88)	(1,367.40, 4,184.50)	(1.00, 5.30)	(0.50, 2.50)	(0.29, 0.88)	(1,367.40, 4,184.50)
7–27 days	1.54	2439.70	5.30	0.80	1.54	2439.70
	(0.83, 2.67)	(1,319.90, 4,227.50)	(1.90, 12.00)	(0.30, 1.90)	(0.83, 2.67)	(1,319.90, 4,227.50)
1–5 months	14.69	3075.20	587.90	12.30	14.63	3064.00
	(8.05, 25.27)	(1,686.10, 5,291.50)	(354.50, 912.10)	(7.40, 19.10)	(8.01, 25.22)	(1,676.90, 5,279.80)
6–11 months	21.07	3588.40	738.00	12.60	21.01	3578.10
	(11.87, 34.65)	(2,021.30, 5,901.10)	(446.20, 1,149.20)	(7.60, 19.60)	(11.79, 34.59)	(2,008.60, 5,892.00)
12–23 months	39.01	2945.10	1500.90	11.30	38.88	2935.50
	(22.12, 66.95)	(1,670.20, 5,054.80)	(903.40, 2,301.40)	(6.80, 17.40)	(22.02, 66.81)	(1,662.10, 5,043.80)
2–4 years	154.05	2910.20	5267.80	10.00	153.60	2901.70
	(90.38, 255.67)	(1,707.40, 4,830.10)	(3,008.10, 8,461.70)	(5.70, 16.00)	(89.93, 255.37)	(1,698.90, 4,824.30)
5–9 years	267.40	2,792.10	6,244.90	6.50	266.79	2,785.80
	(156.26, 425.10)	(1,631.60, 4,438.70)	(3,499.40, 10,057.30)	(3.70, 10.50)	(155.49, 424.40)	(1,623.60, 4,431.50)
10–14 years	231.93	2,690.80	3,889.30	4.50	231.48	2,685.60
	(129.50, 374.56)	(1,502.40, 4,345.60)	(2,184.80, 6,104.30)	(2.50, 7.10)	(128.99, 373.96)	(1,496.50, 4,338.60)
15–19 years	176.27	2,360.60	1,998.20	2.70	175.90	2,355.70
	(100.26, 292.17)	(1,342.60, 3,912.70)	(1,048.90, 3,266.30)	(1.40, 4.40)	(99.87, 291.81)	(1,337.40, 3,907.90)
20–24 years	179.55	2,453.70	1,620.60	2.20	179.24	2,449.50
	(101.50, 301.82)	(1,387.10, 4,124.70)	(825.50, 2,652.60)	(1.10, 3.60)	(101.17, 301.53)	(1,382.60, 4,120.70)
25–29 years	201.45	2,329.40	1,737.40	2.00	201.16	2,326.00
	(112.22, 328.73)	(1,297.60, 3,801.10)	(879.90, 2,792.10)	(1.00, 3.20)	(111.85, 328.45)	(1,293.30, 3,797.90)
30–34 years	266.02	2,195.70	2,259.60	1.90	265.68	2,192.90
	(150.29, 450.06)	(1,240.50, 3,714.80)	(1,254.50, 3,536.20)	(1.00, 2.90)	(149.87, 449.65)	(1,237.00, 3,711.40)
35–39 years	198.72	1,875.40	1,838.20	1.70	198.49	1,873.20
	(111.54, 323.52)	(1,052.60, 3,053.10)	(1,003.30, 2,943.10)	(0.90, 2.80)	(111.31, 323.23)	(1,050.50, 3,050.40)
40–44 years	142.44	1,556.20	1,519.80	1.70	142.31	1,554.70
	(73.70, 231.15)	(805.20, 2,525.30)	(816.30, 2,531.80)	(0.90, 2.80)	(73.59, 231.04)	(804.00, 2,524.00)
45–49 years	139.10	1,260.90	1,812.50	1.60	139.00	1,260.00
	(72.11, 232.80)	(653.60, 2,110.20)	(982.70, 3,065.90)	(0.90, 2.80)	(72.01, 232.73)	(652.70, 2,109.60)
50–54 years	114.08	943.90	1969.20	1.60	114.02	943.40
	(61.70, 188.06)	(510.50, 1,556.10)	(1,089.30, 3,230.70)	(0.90, 2.70)	(61.65, 188.02)	(510.10, 1,555.70)
55–59 years	74.95	681.70	1773.00	1.60	74.92	681.50
	(40.98, 126.44)	(372.70, 1,150.10)	(1,008.60, 2,848.20)	(0.90, 2.60)	(40.95, 126.42)	(372.40, 1,149.80)
60–64 years	36.67	502.30	1173.10	1.60	36.66	502.20
	(20.19, 60.08)	(276.60, 822.90)	(650.70, 1,910.50)	(0.90, 2.60)	(20.18, 60.07)	(276.40, 822.80)
65–69 years	28.48	371.30	1280.90	1.70	28.49	371.40
	(15.35, 45.96)	(200.10, 599.20)	(630.70, 2,151.00)	(0.80, 2.80)	(15.36, 45.96)	(200.30, 599.20)
70–74 years	15.98	299.90	909.60	1.70	15.99	300.00
	(8.71, 26.36)	(163.50, 494.70)	(471.60, 1,503.40)	(0.90, 2.80)	(8.74, 26.36)	(163.90, 494.70)
75–79 years	7.63	230.50	528.60	1.60	7.64	230.70
	(4.20, 12.45)	(126.90, 375.90)	(300.00, 866.50)	(0.90, 2.60)	(4.23, 12.45)	(127.60, 376.00)
80–84 years	4.65	235.10	282.60	1.40	4.66	235.30
	(2.59, 7.89)	(130.80, 398.70)	(157.30, 449.50)	(0.80, 2.30)	(2.60, 7.90)	(131.20, 399.00)
85–89 years	2.31	242.00	119.00	1.20	2.31	242.10
	(1.22, 4.01)	(127.80, 421.00)	(64.30, 193.70)	(0.70, 2.00)	(1.22, 4.01)	(127.90, 421.10)
90–94 years	0.75	256.50	29.80	1.00	0.75	256.60
	(0.36, 1.34)	(122.60, 458.50)	(15.30, 51.90)	(0.50, 1.80)	(0.36, 1.34)	(122.70, 458.60)
95 + years	0.18	285.30	5.70	0.90	0.18	285.30
	(0.09, 0.33)	(137.60, 518.90)	(2.40, 10.80)	(0.40, 1.70)	(0.09, 0.33)	(137.70, 518.80)

ASIR stands for Age-Standardized Incidence Rate; ASPR refers to Age-Standardized Prevalence Rate; ASR-DALYs indicates Age-Standardized Disability-Adjusted Life Year Rate.

Compared to 1990, the disease burden of VAD for both males and females has significantly decreased, but the overall disease burden of VAD in females is higher than in males. In 2021, the number of new cases, ASIR, and the number of prevalent cases, ASPR in females were all higher than in males, but the DALYs and ASR-DALYs in males were higher than in females (Table 2).

### 3.3 Prediction of VAD disease burden in China from 2022 to 2050

The predictions based on the Bayesian Age-Period-Cohort (BAPC) model indicate that from 2022 to 2050, the Age-Standardized Disability-Adjusted Life Year Rate (ASR-DALYs) for Vitamin A Deficiency (VAD) in China will tend to stabilize. The overall population rate will slowly decrease from 3.17 per 100,000 in 2022 to 2.70 per 100,000 by 2050. For males, it will decrease from 3.40 per 100,000 in 2022 to 2.92 per 100,000 in 2050, and for females, it will decrease from 2.95 per 100,000 in 2022 to 2.61 per 100,000 in 2050.

**Figure 4:**
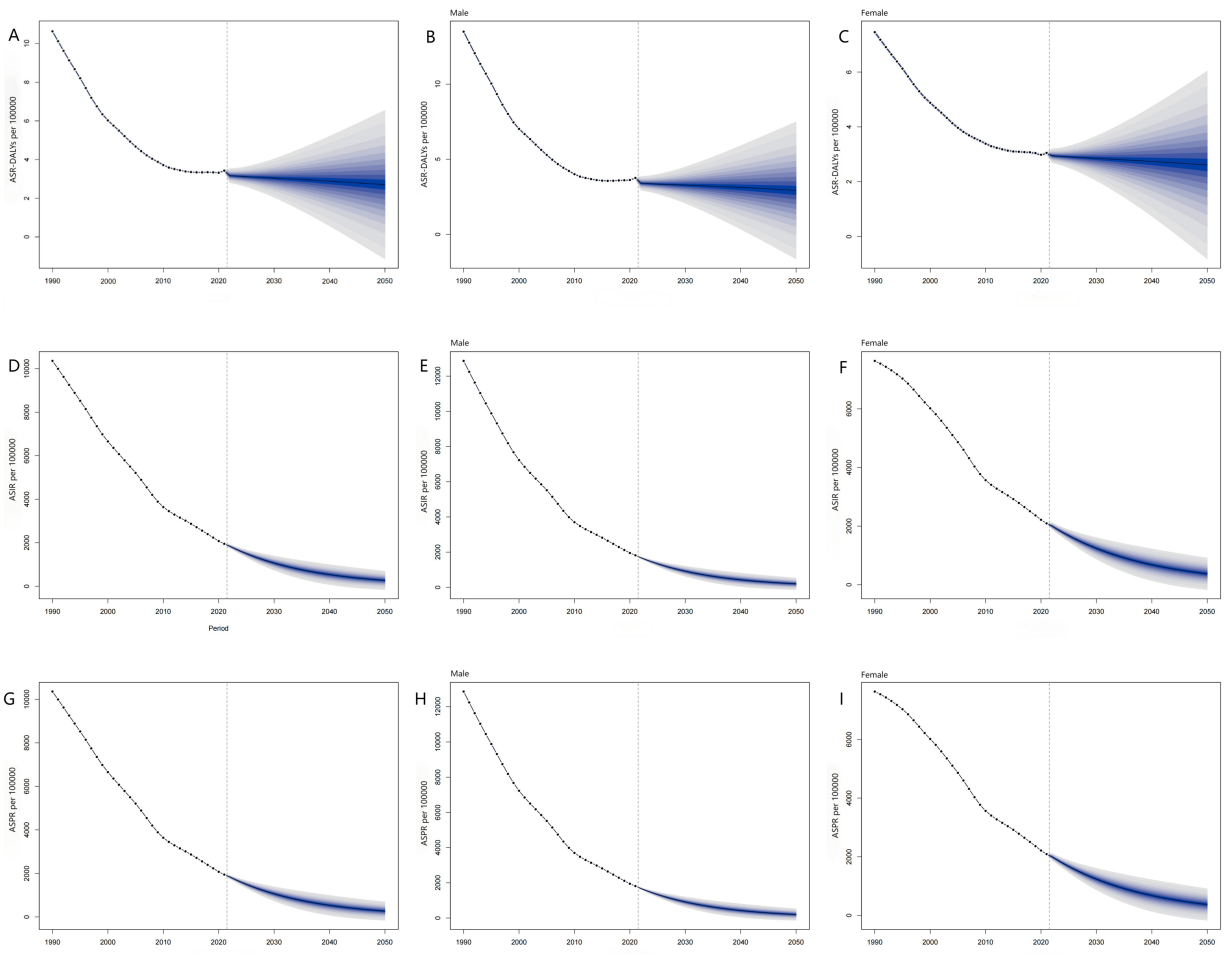
Prediction of vitamin A deficiency disease burden in China from 2022 to 2050. **(A)** Prediction of Age-Standardized DALY Rate; **(B)** Prediction of Age-Standardized DALY Rate in Male; **(C)** Prediction of Age-Standardized DALY Rate in Female; **(D)** Prediction of Age-Standardized Incidence Rate; **(E)** Prediction of Age-Standardized Incidence Rate in Male; **(F)** Prediction of Age-Standardized Incidence Rate in Female; **(G)** Prediction of Age-Standardized Prevalence Rate; **(H)** Prediction of Age-Standardized Prevalence Rate in Male; **(I)** Prediction of Age-Standardized Prevalence Rate in Female.

Both the Age-Standardized Incidence Rate (ASIR) and Age-Standardized Prevalence Rate (ASPR) show a continuous downward trend. The overall population ASIR will drop from 1,838.15 per 100,000 in 2022 to 269.57 per 100,000 by 2050, while for males, it will decrease from 1,678.85 per 100,000 in 2022 to 200.16 per 100,000 in 2050, and for females, it will decrease from 1,999.87 per 100,000 in 2022 to 373.74 per 100,000 in 2050. The overall population ASPR will fall from 1,835.08 per 100,000 in 2022 to 267.89 per 100,000 in 2050, for males, it will drop from 1,675.08 per 100,000 in 2022 to 198.25 per 100,000 in 2050, and for females, it will decrease from 1,997.78 per 100,000 in 2022 to 372.67 per 100,000 in 2050 (Figure 4).

## 4 Discussion

This study, based on the Global Burden of Disease database, analyzes the dynamic changes in the disease burden of Vitamin A Deficiency (VAD) in China from 1990 to 2021 and predicts the disease development trend from 2022 to 2050. The research results provide a scientific basis for public health policy makers, helping to optimize resource allocation and develop targeted intervention measures. The study shows that from 1990 to 2021, the number of new cases of VAD in China (−81.83%), the number of prevalent cases (−81.85%), Disability-Adjusted Life Years (DALYs) (−67.30%), Age-Standardized Incidence Rate (ASIR) (−81.17%), Age-Standardized Prevalence Rate (ASPR) (−81.20%), and Age-Standardized Disability-Adjusted Life Year Rate (ASR-DALYs) (−67.92%) all significantly decreased. This trend aligns with the global decline in the burden of nutrition-related deficiencies (17), yet the magnitude of reduction significantly surpasses the average level observed in other developing countries (13). This may be attributed to the synergistic effects of China’s unique nutritional improvement programs (18, 19) and rapid socio-economic development. It is worth noting that after 2010, the DALYs indicator showed a stable trend, which may indicate that the effect of existing intervention plans on severe cases of VAD has approached the saturation level, highlighting the new challenges faced by disease prevention and control work in the critical stage.

Vitamin A (retinol), as a fat-soluble vitamin, plays a key regulatory role in various physiological processes, including but not limited to cell proliferation and differentiation, immune homeostasis maintenance, circadian rhythm regulation, visual phototransduction, and reproductive system development, and other biological functions (20–23). Epidemiological studies have revealed significant etiological differences in vitamin A deficiency (VAD) across regions with varying economic development levels: In high-income countries, VAD primarily occurs secondary to functional disorders or organic pathologies affecting the entero-hepatic-pancreatic axis, or secondary to neurodevelopmental disorders such as autism spectrum disorders. In contrast, in resource-limited settings, the primary pathophysiological basis of VAD consists of chronic nutritional insufficiency due to inadequate dietary intake combined with persistent low-grade inflammation induced by recurrent gastrointestinal infections (24–26). This regional difference reflects the significant association between VAD as a nutritional deficiency disease and the level of socio-economic development.

In recent years, the global burden of VAD has shown a significant improvement trend (27), however, the disease burden of VAD in China in 2021 remains high. Data analysis found that the disease burden in the female population is generally higher than in males, which is consistent with previous domestic studies (28). This gender difference may stem from multi-level biological and behavioral mechanisms:

(1) Dietary restrictions due to weight control behaviors in females directly affect the bioavailability of retinol (28). Estrogen significantly induces the expression of retinol-binding protein mRNA in the kidney through a nuclear estrogen receptor-mediated mechanism, which may affect the distribution kinetics of vitamin A (29).
(2) Additionally, blood volume expansion during pregnancy leading to hemodilution and fetal development consuming maternal vitamin A reserves; as well as the transfer amount of vitamin A during lactation being 60 times the placental transfer amount during pregnancy (30, 31), together forming the physiological basis for the higher risk in females.

Males have higher DALYs and ASR-DALYs than females, indicating that males may face higher risks in terms of health loss due to VAD. Future VAD prevention and control strategies need to pay further attention to gender differences to optimize intervention measures and reduce the disease burden.

In age groups, the ASIR, ASPR, and ASR-DALYs of VAD show an increasing and then decreasing trend, all peaking in the 6–11 month age group. This study result verifies that age is a key risk factor in the occurrence and development of VAD, indicating that this age group is a high-risk period for VAD, reflecting the nutritional transition challenges during the weaning period and the decline in vitamin A content of breast milk, necessitating enhanced monitoring and intervention during this critical window period. According to WHO guidelines (32), children between 6–11 months should receive an initial vitamin A supplement dose of 100,000 IU, followed by maintenance doses of200,000 IU administered at 4–6 month intervals until reaching 59 months of age, with dosage modifications guided by regular serum retinol concentration assessments. The ASIR and ASPR of the 12–23 month age group show the most significant downward trend, while the ASR-DALYs of the 0–6 day age group show the greatest decrease, indicating that the early infancy period is a critical time for VAD prevention and control, and early intervention measures (such as the use of vitamin A supplements) may play an important role in reducing the disease burden (33). Children under 5 years old are a high-incidence population for VAD, and their pathophysiological mechanism involves a vicious cycle: on one hand, recurrent infections accelerate the consumption of liver vitamin A reserves through pro-inflammatory cytokines; on the other hand, vitamin A deficiency further increases susceptibility to infections by damaging mucosal barrier function and immune function (20, 25, 34–37). This “infection-malnutrition” synergistic mechanism not only directly affects children’s growth and development indicators but may also produce long-term health and economic impacts through pathways such as epigenetic modifications. Studies have found that ASIR, ASPR, and ASR-DALYs decrease with age, but the number of new cases and prevalent cases in adults increases, which may be attributed to China’s focus on the growth and development, nutritional health, and aging issues of adolescents and children. While continuously strengthening interventions for key populations such as children (especially infants and young children) and women of childbearing age, there is an urgent need to establish a comprehensive vitamin A monitoring and supplementation system throughout the life cycle.

Based on the BAPC model prediction analysis, the evolution of the disease burden of VAD in China from 2022 to 2050 will show a differentiated trend: ASR-DALYs will enter a plateau phase, while ASIR and ASPR will continue to decline. This forecast indicates that although the disease burden of VAD will continue to decrease in the coming decades, completely eliminating VAD still faces challenges. To further reduce the disease burden, it is necessary to break through the limitations of traditional prevention and control models, integrate precise intervention strategies (including targeted management of high-risk populations, optimized supplement dosing regimens, and evidence-based nutritional education) with environmental intervention measures such as food fortification, and build a multi-dimensional comprehensive prevention and control system (38–41). At the same time, it is necessary to deeply explore the biological mechanisms and social determinants of the plateau phase to provide a scientific basis for formulating the next stage of elimination strategies.

This study has certain limitations: the GBD data is based on model calculations and there is a certain deviation from actual data. The assessment of the study is limited to the national level of disease burden and does not delve into the detailed analysis of provincial-level differences, urban–rural differences, economic factors, and policy changes. Moreover, since the GBD survey is based on the national level, its results may carry the risk of ecological bias when applied to China’s diverse regional characteristics.

## 5 Conclusion

From 1990 to 2021, the disease burden of Vitamin A Deficiency (VAD) in China has shown an overall downward trend; however, the burden remained relatively high in 2021. Additionally, the disease burden was found to be greater in females than in males, and higher in children than in adults. Future strategies for the prevention and control of VAD should be tailored to the epidemiological characteristics of VAD in China, placing emphasis on interventions targeting females and children. Concurrently, it is essential to enhance public health education for the entire population and to develop and implement effective public health prevention policies. These measures aim to further reduce the disease burden of VAD and promote the achievement of the strategic goal of eliminating micronutrient deficiency disorders as outlined in the “Healthy China 2030” planning outline.

## Data Availability

Publicly available datasets were analyzed in this study. This data can be found here: https://figshare.com/articles/dataset/Vitamin_A_Deficiency/29940080/1?file=57280847.

## Footnotes

^1^ https://vizhub.healthdata.org/gbd-results/
